# Mass Change of Glaciers in Muztag Ata–Kongur Tagh, Eastern Pamir, China from 1971/76 to 2013/14 as Derived from Remote Sensing Data

**DOI:** 10.1371/journal.pone.0147327

**Published:** 2016-01-20

**Authors:** Zhen Zhang, Shiyin Liu, Junfeng Wei, Junli Xu, Wanqin Guo, Weijia Bao, Zongli Jiang

**Affiliations:** 1 State Key Laboratory of Cryospheric Sciences, Cold and Arid Regions Environmental and Engineering Research Institute, Chinese Academy of Sciences, Lanzhou, Gansu, China; 2 University of Chinese Academy of Sciences, Beijing, China; 3 School of Architecture and Urban Planning, Hunan University of Science and Technology, Xiangtan, Hunan, China; 4 Hunan Province Key Laboratory of Coal Resources Clean-Utilization and Mine Environment Protection, Hunan University of Science and Technology, Xiangtan, Hunan, China; Institute of Tibetan Plateau Research, CHINA

## Abstract

The assessment of glacier mass budget is crucial for assessing water reserves stored in glaciers. Derived glacier mass changes in the Muztag Ata and Kongur Tagh (MAKT) region in the eastern Pamir, northwestern China, is helpful in improving our knowledge of the dynamics of glaciers under a changing climate in High Mountain Asia. Here, glacier area and mass changes derived from remote sensing data are investigated for the period 1971/76–2013/14 for glaciers in MAKT. We have used ASTER images (2013/14), Cartosat-1 (2014) and Landsat, SRTM (Shuttle Radar Terrain Mission) digital elevation model (DEM) (2000), topographic maps (1971/76) and the first and second Chinese glacier inventories (CGIs). Our results indicated that the glacier area of MAKT decreased from 1018.3 ± 12.99 km^2^ in 1971/76 to 999.2 ± 31.22 km^2^ in 2014 (–1.9 ± 0.2%). Weak area shrinkage of glaciers by 2.5 ± 0.5 km^2^ (0.2 ± 0.1%) happened after 2000 and the period 2009–2014 even saw a slight expansion by 0.5 ± 0.1 km^2^ (0.1 ± 0.0%). The glaciers in this region have experienced an overall loss of –6.99 ± 0.80 km^3^ in ice volume or –0.15 ± 0.12 m water equivalent (w.e.) a^–1^ from 1971/76 to 2013/14. The mass budget of MAKT was –0.19 ± 0.19 m w.e. a^−1^ for the period ~1971/76–1999 and –0.14 ± 0.24 m w.e. a^−1^ during 1999–2013/2014. Similar to previous studies, there has been little mass change in the Pamir over recent decades despite such uncertainties. Glacier mass change showed spatial and temporal heterogeneity, with strong mass loss on debris-covered glaciers with an average of –0.32 ± 0.12 m w.e. a^−1^ from the 1970s to 2013/14.

## Introduction

Glaciers are sensitive to climate change and play an important role in the discharge of many Asian rivers [1]. In past decades, many mountain glaciers have progressively shrunk in mass and extent in response to global warming [2]. However, slight mass gains or balanced mass budgets have been reported for several glaciers in recent years, especially in central Karakoram [3], western Pamir [4], and West Kunlun Shan [5–7]. In addition, there has been recent debate on whether the mass budget of glaciers in the Pamir is currently in balance. Gardner, Moholdt [8] and Kääb, Treichler [9] found presumably negative mass budgets in Pamir using ICESat GLAS (Geoscience Laser Altimeter System) data from ICESat, but their results are different with slight mass loss of 0.13 ± 0.22m w.e. a^−1^ during 2003–2009 by Gardner, Moholdt [8] and a large mass loss of 0.48 ± 0.14 m w.e. a^–1^ by Kääb, Treichler [9] over the Pamir. However, a positive balance of +0.14 ± 0.13mw.e.a^−1^ was found by Gardelle, Berthier [4] in the western Pamir by comparing the 2000 SRTM (Shuttle Radar Topography Mission) with a 2011 DEM (digital elevation model) derived from SPOT5 stereo imagery. Yao, Thompson [10] reported a positive budget of 0.25 m w.e. a^−1^ from 2005/2006 to 2009/2010 for the Muztag Ata Glacier (approximately 1 km^2^, 5235–5940m a.s.l.) on the southern slope of Muztag Ata. Continued measurements based on additional observations above 5700 m a.s.l. showed less positive values of +0.05 m w.e. a^−1^ for 2010/2011 to 2013/2014 [11]. Holzer, Vijay [11] used Hexagon KH-9, ALOS-PRISM, Pléiades, and SRTM-3 DEM data and found average mass changes in the range of −0.03 ± 0.33 m w.e. a^−1^ (1973–2009) to −0.01 ± 0.30 m w.e. a^−1^ (1973–2013), which revealed nearly balanced budgets at Muztag Ata in eastern Pamir for the last 40 years.

Heterogeneous mass wastage has been found across the Pamirs, although the glacier area change was at almost the same level. In western Pamir (Tajikistan), a glacier area decrease of 18.4% was found in the Saukdara and Zulumart Ranges during 1978–2001 [12], increased by 3.46% from 1992 to 2006 in the Fedchenko basin [13], and decreased by 1.4% in the Fedchenko Glacier during 1928–2007 [14]. In eastern Pamir (China), Cai, Ma (15) compared ASTER images of the Muztag Ata area (acquired in 2001) with topographical maps corrected by aerial photographs (acquired in 1965) and found that the glacier area had decreased by approximately 1.11% while part of the glaciers in the west and south of the Muztag Ata have increased from 1965 to 2001; Shangguan, Liu [16] used aerial photographs (1962/66) and Landsat TM (1990) and ETM+ (1999) images and found that the glacier area decreased by 7.9%, mainly due to changes observed in the most recent period (1990–99), when the annual area loss almost tripled to 1.01 km^2^ a^–1^ in Muztag Ata and Kongur Tagh (MAKT). Holzer, Vijay [11] showed that glaciers in Muztag Ata were shrinking by –0.6 ± 3.9% during 1973–2013 with a balance state of mass in the same period. The most recent result for the whole eastern Pamir based on the modified first glacier inventory and a second [17, 18] indicated that the area of glaciers in the region has decreased by 10.8 ± 1.1% during 1963–2009 and glaciers in MAKT were generally retreating (approximately –4%) [19].

There are 1 265 glaciers with a total area of 2054.0 ± 75.9 km^2^ in the eastern Pamir in Chinese territory [19]. Up to now, there is a lack of data for mass changes in glaciers in the region except for that by Holzer, Vijay [11] mentioned above. We will focus on glaciers about ~1000 km^2^ in the glacierized center of MAKT to understand the status of glaciers in the region with different additional data. The objectives of this study are summarized as follows:

1. To update the glacier area change on decadal scale with new boundary data for glaciers in 2014, 2000, and 1971/76.
2. To determine the mass change in different periods in MAKT using geodetic methods based on topographical maps (1971/76), SRTM DEM (2000), ASTER DEM (2013/14), and Cartosat-1 DEM (2014).
3. To analyze the response of debris-covered glaciers in this region.

## Study Area

**Fig 1:**
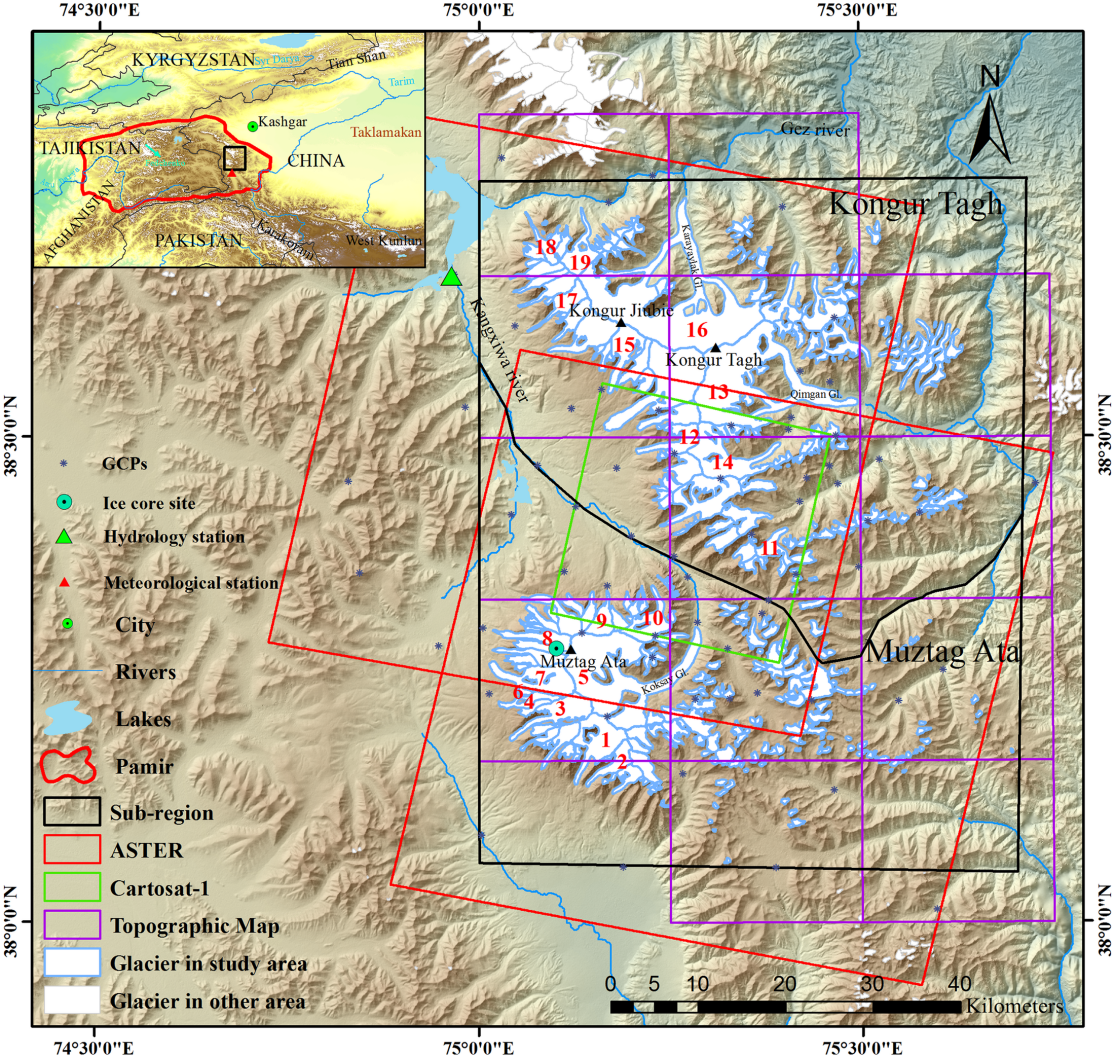
Location and topography of Muztag Ata and Kongur Tagh.

Muztag Ata and Kongur Tagh (MAKT) are located in eastern Pamir, China (38–39° N, 74° 40′–75° 40′ E), with altitudes ranging from 3000 m above sea level (a.s.l.) to maxima of 7546 and 7719 m above sea level (a.s.l.) at the two summits, respectively (Fig 1). Both massifs are ~2000 m higher than any of the neighboring peaks and represent areas of significantly anomalous topography [20, 21]. Several valley glaciers descend from the ice caps, most of them with snowline elevations of 5200–5700 m a.s.l. and terminus positions at 3900–4900 m a.s.l. [16, 22], which are of the extreme continental type, and accumulate snow mostly in winter [11, 23, 24]. Muztag Ata and Kongur Tagh are climatologically situated at the intersection of the Indian summer monsoon and mid-latitude westerlies [21]. The highest precipitation occurs between April and May as a result of the penetration of the mid-latitude westerlies into this region [21, 25]. The data collected at the Taxkorgan meteorological station (37° 46′ N, 75° 14′ E; 3090.9 m a.s.l.), which is the only station in eastern Pamir above 3000 m a.s.l., indicated an annual precipitation (1957–2010) of 74.39 mm and a mean summer temperature (June–August) as high as 15.14°C [26]. An ice core with a depth of 41.6 m from Muztag Ata Peak (7010 m a.s.l.) indicated an annual accumulation of 605 mm from 1955–2003 with a maximum of 1390mm in 1971 [27], and decreased precipitation tendency from the 1960s to 2003, while annual precipitation has increased slightly according to observation at the Taxkorgan meteorological station [16, 26]. However, a rapid warming (+2.0 to +2.4°C/10 a) at Muztag Ata as derived from the ice core isotope record while a warming of +0.18°C/10a at the Taxkorgan meteorological station was shown from the 1990s [28]. From July 4 to August 8 in 2001, an observation of glacier ablation on the terminus of the Yangbark Glacier (also called Kematulejia Glacier, No. 9 in Fig 1) on the western side of Muztag Ata (4460–4600 m a.s.l.) also demonstrates the warming trend and the glacier ablation continuously increased compared with the observations in 1960 and 1987 [29]. Glaciers’ meltwater in this region drains to three rivers: the Taxkorgan River, which is a tributary of the Yarkant river, and two tributaries of the Kaxgar river, i.e., the Kusan and Gez rivers.

## Data and Methods

### Topographic maps

Thirteen topographic maps with a 1:50,000 scale were obtained, which were constructed from aerial photogrammetry acquired in the 1970s by the Chinese Military Geodetic Service (CMGS) (Table 1). The geographic projection of the topographic maps was based on the Beijing Geodetic Coordinate System 1954 (BJ54) geoid and the Yellow Sea 1956 datum (the mean sea level at the Qingdao Tidal Observatory in 1956). The contour lines of digital elevation were generated from topographic maps by the State Bureau of Surveying and Mapping of China (SBSMC) and were georeferenced into WGS84/EGM96 using a seven-parameter transformation method and interpolated into DEMs (hereafter, these DEMs are referred to as TOPO DEMs) with a spatial resolution of approximately 30 m by employing the Thiessen polygon method [30]. The error of the coordinate conversion parameters, based on a seven-parameter spatial transformation model, is <0.002 m [31]. The nominal vertical accuracies of these topographic maps were controlled within 3–5 m for the flat (with slopes <2°) and hilly areas (with slopes 2–6°) and controlled within 8–14 m for mountain (with slopes 6–25°) and high mountain areas (with slopes >25°) according to the national photogrammetrical standard issued by the Standardization Administration of the People’s Republic of China (SAC) [32].

**Table 1:** List of Muztag Ata and Konggur Tagh datasets.

Data	Time	Pixel size (nadir, m)/Scale	Purpose	Coverage area
*Input*				
Topographic maps (1971)	Oct 1971	1:50 000	DEM/Glacier outline	Konggur Tagh
Topographic maps (1976)	Nov 1976	1:50 000	DEM/Glacier outline	MAKT
SRTM 1	Feb 2000	30	DEM	MAKT
Terra Aster	11 Jun 2013	15	DEM	Konggur Tagh
Terra Aster	23 Jul 2014	15	DEM	Muztag Ata
Cartosat-1	24 Sep 2014	2.5	DEM	Konggur Tagh
Landsat ETM+	11 Sep 2000	30	Glacier outline	All
Landsat OLI	3 Oct 2014	15	Glacier outline	All
*Reference*				
The first Chinese glacier inventory	1963		Glacier outline	All
The second Chinese glacier inventory	2009		Glacier outline	All
Landsat MSS	Sep 1972	79	Glacier outline	All
Landsat OLI	Sep 2014	15	Glacier outline	All
Landsat OLI	9 Oct2013	15	Glacier outline	All

### SRTM

The SRTM data were obtained during the single-pass interferometric synthetic aperture radar SRTM mission during an 11-day mission in February 2000, in which DEMs can be seen as representative of the glacier surface at the end of the 1999 ablation period with slight seasonal variances [4, 33–37]. The spatial resolution of 1 arc-second (SRTM1) and 3 arc-seconds (SRTM3) is freely available for scientific purposes (http://earthexplorer.usgs.gov/). The SRTM 1 Arc-second global (approximately 30 meters) dataset was released in phases starting from September 24, 2014. The SRTM 1 data offer worldwide coverage of void-filled data at a resolution of 1 arc-second (30 meters). However, some tiles may still contain voids. The SRTM1 data in our study contains voids, especially at high and steep elevation regions due to radar shadows and layover effects, similar to the unfilled finished SRTM 3 data (90 m) [35]; the coordinates are WGS84/EGM96 [33]. Thus, parts of the accumulation regions are not covered by the SRTM1 DEM. The SRTM void-filled elevation data (1 arc-second for the United States and 3 arc-seconds for global coverage) were also provided by the U.S. Geological Survey (USGS), and the gaps were filled by the Consultative Group for International Agricultural Research (CGIAR) using auxiliary data [38]. Because we do not know the exact time of the filled data, unfilled SRTM1 data were used in our study. The accuracy of SRTM3 is specified as 16 m with a 90% confidence level [39, 40]. There is a relative difference of ±13.5 m (root-mean square error, RMSE) between SRTM and TOPO DEM (1:50 000) [41]. A comparison of SRTM1 and unfilled SRTM3 data (resampled into a spatial resolution of 30 m) for our study area (DEM coregistration method cf. section 3.7, shift X of –0.07 m and shift Y of 3.43 m on horizontal displacements between DEMs) indicated a mean difference value of –0.01 m (standard deviation: 4.66 m). Thus, SRTM1 data can also be used to calculate elevation changes with high resolution.

However, the penetration of the C-band radar waves needs to be taken into account for volume change as it varies from 0 to 10 m depending on the local climatic conditions [3, 34, 39, 42]. A Landsat ETM+ scene from 7 February, which is near the time of the SRTM mission (11–20 February 2000), revealed slight snow coverage with mostly snow-free glacier tongues in the study area. The comparison between SRTM X-band and SRTM C-band was used to estimate and correct the C-band radar penetration into snow and ice by assuming that the penetration depth of the X-band signal is negligible for glaciers in our study area, which has a higher temperature and is less dry than the Antarctic ice sheet [43]. Because of incomplete spatial coverage (only 20% of our study area) by the SRTM X-band data, it is not used in the first place to compute elevation changes. To separate between accumulation and ablation areas, we assumed the equilibrium line altitude (ELA) of each glacier to be approximately equal to the median glacier altitude, which is based on the CGIs [44]. When compared with the X-band SRTM (DEM coregistration method cf. 3.7), the penetration depth of the C-band was averaged as 2.41 m for firn and snow (accumulation zone) and 0.79 m for clean ice ablation zones, assuming no penetration in the case of supraglacial debris.

### ASTER images

The ASTER sensor mounted on the Terra satellite platform provides multispectral imagery between 82° N and 82° S [45]. There are three bands in the visible and near infrared spectral range (VNIR, 0.5–1.0 μm) with a 15-m spatial resolution, six bands in the shortwave infrared spectral range (SWIR, 1.0–2.5 μm) with a 30-m resolution, and five bands in the thermal infrared spectral range (TIR, 8–12 μm) with a 90-m resolution [46]. In the VNIR, one nadir-looking (3N, 0.76–0.86 μm) and one backward-looking (3B, 27.7° off-nadir) camera generate an along-track stereo pair with a base-to-height (B/H) ratio of about 0.6, which is close to ideal for generating DEMs for a variety of terrain conditions via automated techniques [47]. Because of the 15-m resolution of the ASTER 3N and 3B stereo data, the resolution of ASTER DEMs is restricted to comparatively low levels of 30–45 m [48–50]. ASTER DEMs presented accuracies ranging from 7 to 20 m [49, 50].

### Cartosat-1

Cartosat-1 (IRS-P5) was launched by the Indian Space Research Organisation (ISRO) in 2005 and is equipped with two panchromatic cameras tilted at –5° (AFT) and +26° (FWD) from the pitch axis [51, 52]. These satellite cameras have a resolution of 2.5 m and a B/H ratio of 0.62 [53]. Quasi-simultaneous image acquisition is guaranteed by a time lag, similar to the ASTER stereo images, of about 52 seconds between both records, thus facilitating DEM generation [54]. Data from Cartosat-1 are 10 bit and provide rational polynomial coefficients (RPCs) for photogrammetric processing and extraction of 3-D information using a rational function model (RFM). The RPCs have a much lower accuracy than the ground resolution, i.e., approximately 2.5 m. Subpixel accurate ground control points (GCPs) have been used in previous studies to estimate bias or affine RPC correction parameters required for high-quality geolocation of high-resolution satellite images [55]. Cartosat-1 data have been used to glacier mapping [56, 57] and geodetic mass balances [58].

### Chinese Glacier Inventory datasets and temporal glacier boundary mapping

The first and second Chinese glacier inventory (CGI) datasets [18, 59] provided the glacier boundary in 1963 and 2009 for this study [19]. However, the TOPO DEMs were derived from topographic maps constructed in 1971/1976, and glacier outlines were digitized manually. The glacier outlines of the first CGI, which was verified using aerial images [19] and Landsat MSS images, which were taken in 1972/73, were used to examine and correct errors of glacier outlines from topographic maps. The Landsat images (Table 1) were also used to extract manually the glacier boundary in 2000 and 2014 (cf. [18, 60, 61]). In addition, ASTER and Cartosat-1, which were ortho-rectified using the DEMs generated from their own stereo data, were used to check the glacier boundaries in 2000 and 2014 by cross-checking with Google Earth^™^. The hillshade based on the SRTM3 DEM, TOPO DEMs and the calculated ASTER and Cartosat-1 DEMs provided additional information for detection of the glacier boundary. We estimated the uncertainty using a buffer of 6 m for topographic maps with a scale of 1:50 000 (cf. [19, 61]) and half a pixel for the Landsat images. We assumed that the uncertainty due to image coregistration was captured using the buffer method [35, 62–64]. Considering the law of error propagation, the final uncertainty for area change (*E*_AC_) was calculated using Eq (1):

$$E_{AC}=\sqrt{{\left(E_{A1}\right)}^{2}+{\left(E_{A2}\right)}^{2}},$$ (1)

where *E*_A1_ and *E*_A2_ represent the uncertainties of the glacier outlines; however, the same borderlines were removed in periods 1 and 2.

### DEM generation and postprocessing

ASTER and Cartosat-1 were processed using the “DEM Extraction” Model of the ENVI 5.0 software using the geographic reference system WGS84 UTM zone 43 N. During the DEM generation, at least 20 GCPs for each stereo image (Fig 1) were identified from the topographic maps and Landsat images on ice-free terrains as the elevation and horizontal references, respectively. The automatically generated tie points (TPs) were visually checked via ground objects and topographic features. At least 60 TPs for each scene were selected to maintain an even distribution of TPs in an image, especially TPs in snow, shadows, and other areas, to increase the accuracy of image-to-image coregistration. The maximum Y parallax errors were controlled to less than 0.5–0.8 pixels in our study. The spatial resolution of the ASTER DEM was 30 m and the spatial resolution of the Cartosat-1 DEM was 10 m. However, all of the DEMs were resampled to a spatial resolution of 30 m by cubic convolution. The DEMs from the ASTER and Cartosat-1 image pairs exhibited high noise in some glacier areas with snow and shadows, and these outliers were deleted. The DEMs and the region covered by these DEMs are shown in Table 2.

**Table 2:** Glacier areas covered by DEMs for mass change investigation.

Reg.	Item	Altitude zone (m a.s.l.)	Glaciers area covered by DEM (km^2^)	Percentage of total glacier area (100%)
**Muztag Ata**	SRTM—TOPO	3900–7600	346.6	87.7
	ASTER—SRTM	3900–7600	326.0	82.5
	ASTER—TOPO	3900–7600	277.5	70.2
**Kongur Tagh**	SRTM—TOPO	2700–7600	638.1	100
	ASTER—SRTM	2700–7600	273.0	42.8
	ASTER—TOPO	2700–7600	273.0	42.8
	Cartosat-1—SRTM	3800–6500	82.0	12.8
	Cartosat-1—TOPO	3800–6500	82.0	12.8

### DEM Coregistration and Accuracy

Relative horizontal and vertical biases are often observed during matching of multisource (or temporal) DEMs [65, 66]. These biases may occur due to technique limitations, sensor inconstancy, and inappropriate conditions at the ground surface [67]. These biases always produce uncertainties when deriving precise changes of glacier thickness or volume, and they can be corrected using the relationship between elevation difference and aspect (cf. [66]). The TOPO DEM, with the highest accuracy and reliability [30, 32, 36, 37], was chosen as the reference DEM to assess the accuracy of other DEMs by comparing the elevation values on the nonglacier terrains. To account for the slope dependency of the method, we excluded all terrain below a slope of 5°. Previous studies have shown that vertical biases are significantly correlated with topographical characteristics, such as aspect, slope, altitude, and maximum curvature [4, 34, 68]. The substantial trigonometric relationship between standardized vertical bias and topographical parameters (slope and aspect) allows for the simultaneous detection of vertical biases and horizontal displacements [66]. The relationship between elevation biases and maximum curvatures both on and off glacierized areas can adjust the biases resulting from multiple spatial resolutions [43]. To consider outliers and curvature effects, we removed the outliers by utilizing the threshold of the 5% and 95% quantiles based on statistics (cf. [34]).

After the adjustments, we calculated statistical parameters for the differences of the nonglacier area between the final adjusted DEMs (Table 3). The mean elevation difference (*MED*) of the nonglacier area between the final adjusted DEMs was in the range of –0.99 to 0.34 m (Table 3). The standard deviation of the nonglacier area (*STDV*_no glac_) would probably overestimate the uncertainty due to averaging over larger regions, which reduces the errors [58, 69]. Thus, the standard error of the mean (*SE*) used for estimation of the uncertainty is given by

$$SE=STDV_{noglac}/\sqrt{N},$$ (2)

where *N* is the number of included pixels. To minimize the influence of autocorrelation, it is recommended that the de-correlation length be chosen based on the spatial resolution. For DEMs with a spatial resolution of 5 m, a de-correlation length of 100 m is suitable [70]. Berthier, Schiefer [69] chose 500 m as the de-correlation length for DEMs with a resolution of 40 m, and Bolch, Pieczonka [58] used values of 600 m for the spatial resolution of 30 m and 400 m for 10–20 m. In our study, a value of 600 m was chosen as the de-correlation length according to the spatial resolution of 30 m. Then, the accuracy (*σ*) was determined using the *SE* and *MED* over the ice-free terrain [58]:

$$\sigma =\sqrt{MED^{2}+SE^{2}}.$$ (3)

**Table 3:** Statistics of original and adjusted vertical errors of the three DEMs.

		Original (m)		Adjusted (m)				
Reg.	Item	MED^a^	STDV^b^	MED	STDV	N^c^	SE^d^ (m)	σ^e^ (m)
**Muztag Ata**	SRTM—TOPO	-11.13	18.79	-0.91	17.32	6329	0.21	0.94
	ASTER—SRTM	-12.41	38.09	-0.76	27.38	1878	0.63	0.99
	ASTER—TOPO	-19.24	34.24	-0.22	28.84	2213	0.61	0.65
**Kongur Tagh**	SRTM—TOPO	-9.13	21.50	-0.99	19.27	6954	0.23	1.02
	ASTER—SRTM	-9.62	54.37	-0.82	31.08	1912	0.71	1.08
	ASTER—TOPO	-7.67	49.45	0.34	38.34	2389	0.78	0.85
	Cartosat-1—SRTM	-12.73	20.30	0.10	18.75	458	0.88	0.88
	Cartosat-1—TOPO	-10.18	28.77	-0.97	26.51	1067	0.81	1.26

^a:^ MED—Mean elevation difference;

^b:^ STDV—Standard deviation;

^c:^ N—Number of considered pixels;

^d:^ SE—Standard error;

^e:^ σ—The accuracy of DEMs difference.

The accuracies for all subregions were calculated for the investigated periods. An accuracy ranging from 0.65 to 1.26 m (Table 3) indicates that the postprocessed DEMs are acceptable and suitable for the estimation of glacier volume changes.

Considering the law of error propagation from one year to the next, the annual mean uncertainty (*ε*) is defined as:

$$\varepsilon =\sigma /\sqrt{n},$$ (4)

where *n* is the number of years for a period.

### Glacier mass balance

Mass change was calculated based on the glacier surface elevation change or volume change using a density assumption or model. Kääb, Berthier [71] proposed three density scenarios to measure the glacier mass change assuming density ranges from 900 to 600 kg/m^3^. Huss [72] suggested a value of 850 kg/m^3^ for the conversion of volume change to mass change, and then added an ice density uncertainty Δ*ρ* of 60 kg/m^3^. In this study, we used a density of 850 ± 60 kg/m^3^ to estimate the mass change in water equivalents (w.e.).

## Results

### Glacier area change

There were 434 glaciers in MAKT with a total area of 1018.3 ± 12.99 km^2^ in 1971/76; however, heterogeneous variations were observed for different periods and different glaciers (Table 4) over the past 40 years. The overall area loss of MAKT was –19.1 ± 2.0 km^2^ over the period from 1971/76 to 2013/14, accounting for 1.9 ± 0.2% of its area in 1971/76. The glacier shrank slightly after 2000 (–2.5 ± 0.5 km^2^, or –0.2 ± 0.1%) and even increased during 2009–2014 (0.5 ± 0.1 km^2^, or 0.1 ± 0.0%) (Table 4). Fifty-eight debris-covered glaciers with an area of 644.3 ± 6.1 km^2^ (total debris area of 120.61 ± 3.5 km^2^) were relatively stable (only decreased by 0.4 ± 0.1%) in the past 40 years. The area change was highly variable between different glaciers, even for those adjacent to each other. Most glaciers shrank slightly or were stable after 2000; however, Kuokuosele Glacier (No. 1) and Kuosikulake Glacier (No. 3) decreased by ~0.3%–1.5% (~0.01% a^–1^–0.06% a^–1^) from 1976 to 2000 and then subsequently advanced (~1% or ~0.04% a^–1^). Glacier G075087E38696N (No. 18) kept advancing throughout the period of investigation and showed rapid change trends, i.e., +26.5 ± 0.6 m a^–1^ from 1976–2000, followed by +43.1 ± 1.6 m a^–1^ from 2000 to 2009, and +79.4 ± 3.0 m a^–1^ from 2009 to 2014 (enlargement of +5.3 ± 1.1%). The three largest glaciers (Karayaylak, Qimgan, and Koksay) are heavily covered by debris and were relatively stable from 1976 to 2014 (Table 4).

**Table 4:** Glacier area (A) and changes at Muztag Ata and Kongur Tagh for selected glaciers with mass-balance estimates, and for all glaciers of the study site.

Reg.	Gla.ID	Glacier (GLIMSID)	A_1963_^a^(km^2^)	A_1971/76_(km^2^)	A_2000_(km^2^)	A_2009_^b^(km^2^)	A_2014_(km^2^)	Area change(1971/76-2014)(km^2^)
**Muztag Ata**	1	G075156E38189N(Kuokuosele Gl.)	20.3±1.32	20.3±1.32	20.3±0.52	20.4±0.53	20.5±0.53	0.1±0.0(0.7±0.1%)
	2	G075179E38166N	8.0±0.10	8.0±0.10	8.0±0.27	8.0±0.27	8.0±0.27	0.0±0.0(0.0±0.0%)
	3	G075112E38219N(Kuosikulake Gl.)	16.4±0.20	16.4±0.19	16.1 ±0.49	16.0±0.48	16.3±0.48	-0.1±0.0(-0.5±0.2%)
	4	G075072E38230N	5.8±0.12	5.8±0.12	5.8 ±0.30	5.8±0.30	5.8±0.30	-0.1±0.0(-1.1±0.2%)
	5	G075181E38255N(Koksay Gl.)	84.8±0.64^c^	81.2±0.59	81.2±1.41	81.2±1.41	81.2±1.41	0.0±0.0(0.0±0.0%)
	6	G075060E38238N(Muztag Ata Gl.)	1.2±0.04	1.1±0.04	1.1 ±0.08	1.1± 0.08	1.1± 0.08	0.0±0.0(-0.4±0.1%)
	7	G075092E38247N(Karaxiong Gl.)	19.9±0.23	19.9±0.23	19.9 ±0.56	19.9±0.56	19.9±0.56	0.0±0.0(0.0±0.0%)
	8	G075086E38293N(Kematulejia Gl.)	9.5±0.13	9.4±0.13	9.3±0.32	9.3±0.32	9.3±0.32	-0.2±0.0(-1.9±0.2%)
	9	G075161E38311N	7.9±0.09	7.9±0.09	7.9 ±0.23	7.9±0.23	7.9±0.23	0.0±0.0(-0.4±0.1%)
	10	G075247E38305N	13.5±0.18	13.4±0.17	13.1±0.41	13.1±0.41	13.1±0.41	-0.3±0.0(-2.6±0.1%)
subtotal			394.5±5.60	385.5±5.59	375.9±13.99	374.9±13.23	375.3±13.25	-4.9±1.5(-2.6±0.4%)
**Kongur Tagh**	11	G075386E38386N	6.9±0.10	6.9±0.10	6.9 ±0.27	6.9±0.27	6.9±0.27	0.0±0.0(0.0±0.0%)
	12	G075267E38499N	23.0±0.28	23.0±0.28	23.0 ±0.77	23.0±0.77	23.0±0.77	0.0±0.0(0.0±0.0%)
	13	G075339E38560N(Qimgan Gl.)	86.8±0.66	86.8±0.66	86.7 ±1.94	86.6±1.64	86.6±1.64	-0.2±0.0(-0.2±0.0%)
	14	G075321E38480N	27.4±0.28	27.4±0.28	26.5 ±0.70	26.5±0.70	26.5±0.70	-0.9±0.0(-3.3±0.1%)
	15	G075191E38590N(Konggurjiubie Gl.)	14.1±0.18	14.1±0.18	14.1±0.44	14.1±0.44	14.1±0.44	-0.1±0.0(-0.4±0.0%)
	16	G075254E38623N(Karayaylak Gl.)	115.3±0.79	115.2±0.81	115.2±1.93	115.2±1.93	115.2±1.93	-0.1±0.0(-0.1±0.0%)
	17	G075116E38641N	8.8±0.14	8.8±0.14	9.0 ±0.32	9.0±0.32	9.0±0.32	0.2±0.0(2.5±0.0%)
	18	G075087E38696N	4.2±0.08	4.2±0.08	4.3 ±0.21	4.4±0.22	4.4±0.24	0.2±0.0(5.3±1.1%)
	19	G075133E38690N	11.6±0.15	11.6±0.15	11.6 ±0.38	11.6±0.38	11.6±0.38	0.0±0.0(-0.4±0.0%)
subtotal			646.1±7.41	632.8±7.40	625.8±18.06	623.8±17.97	623.9±17.99	-8.9±1.3(-1.4±0.2%)
**total**			1040.6±13.01	1018.3±12.99	1001.7±31.33	998.7±31.19	999.2±31.22	-19.1±2.0(-1.9±0.2%)

^a:^ 1963 glacier area data obtained from the first CGI revision;

^b:^ 2009 glacier area data obtained from the second CGI;

^c:^ Koksay glacier cracked and disintegrated, creating a small branch with an area of 2.4 ± 0.2 km^2^.

### Glacier mass change

**Fig 2:**
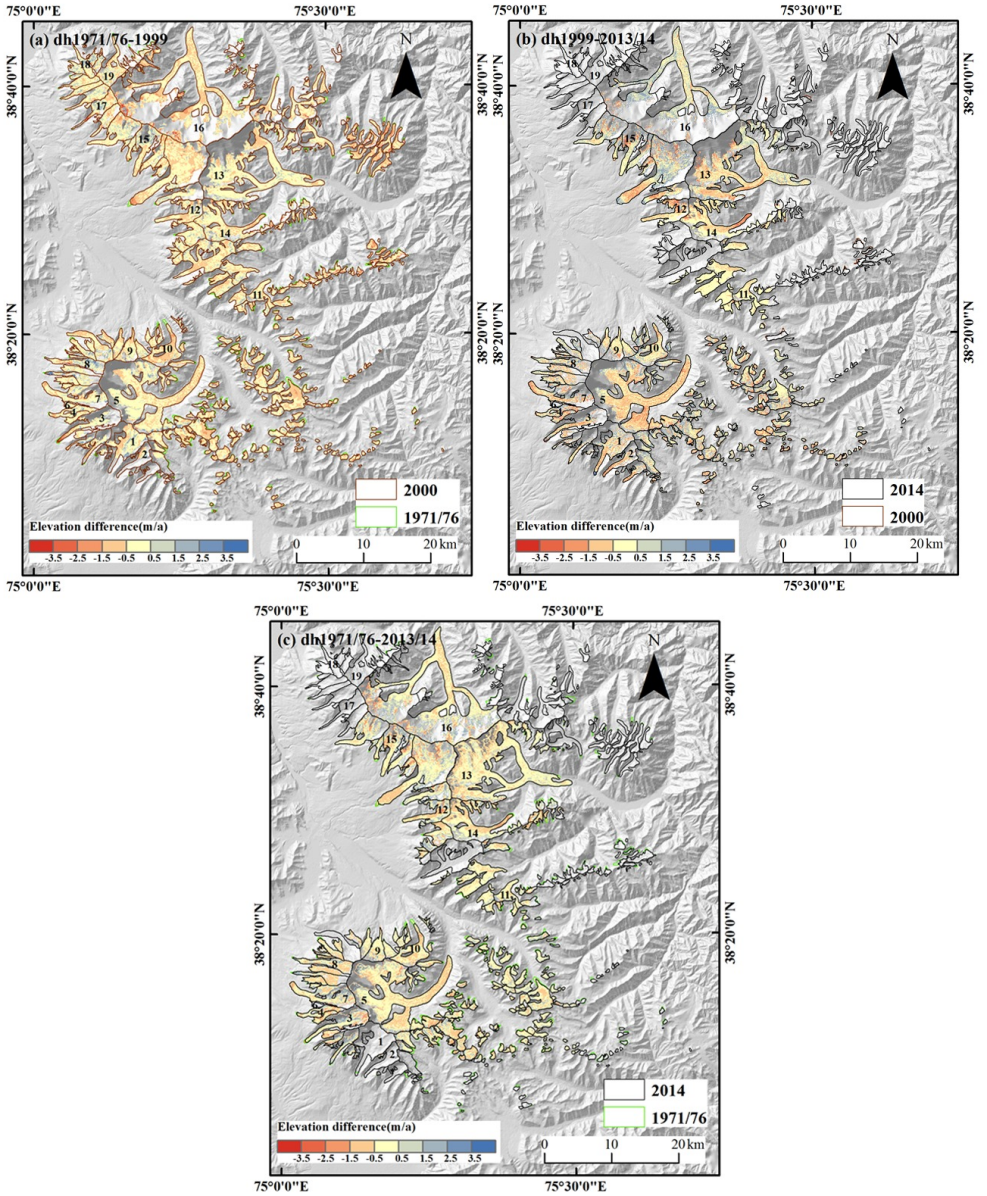
Elevation difference of glaciers in MAKT. (a) Elevation difference between TOPO DEM (1971/6) and SRTM (1999); (b) Elevation difference between SRTM (1999) and ASTER (2013/14); (c) Elevation difference between TOPO DEM (1971/6) and ASTER (2013/14).

Most of the glaciers show a slight surface lowering in the MAKT of –6.86 ± 0.78 m for ~1971/76–2013/14. The glaciers in the MAKT have experienced an overall loss of –6.99 ± 0.80 km^3^ in ice volume or –0.15 ± 0.12 m w.e. a^–1^ from 1971/76 to 2013/14. The overall mass budgets of glaciers in Muztag Ata and Kongur Tagh were –0.15 ± 0.18 and –0.21 ± 0.19 m w.e. a^−1^, respectively, for ~1971/76–1999; –0.13 ± 0.23 and –0.16 ± 0.25 m w.e. a^−1^ during 1999–2013/2014 (Table 5; Fig 2).

**Table 5:** Glacier volume and mass changes during the investigated periods in MAKT

		1971/76-1999		1999-2013/2014		1971/76-2013/2014	
Reg.	Gla.ID	MED^a^ (m)	SMB^b^	MED (m)	SMB	MED (m)	SMB
**Muztag Ata**	1	n.a.^c^	n.a.	0.56±0.99	0.03±0.23	n.a.	n.a.
	2	n.a.	n.a.	-2.77±0.99	-0.16±0.23	n.a.	n.a.
	3	-2.40±0.94	-0.09±0.18	2.85±0.99	0.16±0.23	0.58±0.65	0.01±0.10
	4	0.80±0.94	0.03±0.18	-0.95±0.99	-0.05±0.23	-0.18±0.65	0.00±0.10
	5	-3.70±0.94	-0.13±0.18	-6.04±0.99	-0.34±0.23	-9.96±0.65	-0.22±0.10
	6	1.30±0.94	0.05±0.18	n.c.^d^	n.c.	n.c.	n.c.
	7	-3.08±0.94	-0.11±0.18	0.58±0.99	0.03±0.23	-2.38±0.65	-0.05±0.10
	8	-1.12±0.94	-0.04±0.18	-3.62±0.99	-0.22±0.23	-4.74±0.65	-0.10±0.10
	9	-1.02±0.94	-0.04±0.18	-3.24±0.99	-0.18±0.23	-4.32±0.65	-0.10±0.10
	10	-12.67±0.94	-0.47±0.18	0.22±0.99	0.01±0.23	-11.77±0.65	-0.26±0.10
Average^e^		-4.05±0.94	-0.15±0.18	-2.29±0.99	-0.13±0.23	-6.47±0.65	-0.14±0.10
**Kongur Tagh**	11	-5.35±1.02	-0.20±0.19	-4.90±0.88	-0.28±0.21	-9.34±1.26	-0.20±0.19
	12	-3.61±1.02	-0.13±0.19	-3.52±0.88	-0.20±0.21	-7.20±1.26	-0.16±0.19
	13	-1.22±1.02	-0.05±0.19	-5.07±1.08	-0.31±0.25	-5.78±0.85	-0.13±0.13
	14	-4.45±1.02	-0.16±0.19	-3.49±0.88	-0.20±0.21	-7.96±1.26	-0.18±0.19
	15	-2.03±1.02	-0.07±0.19	-0.20±1.08	-0.01±0.25	-2.26±0.85	-0.05±0.13
	16	-8.79±1.02	-0.32±0.19	6.16±1.08	0.37±0.25	-2.87±0.85	-0.07±0.13
	17	-3.90±1.02	-0.14±0.19	n.c.	n.c.	n.c.	n.c.
	18	4.82±1.02	0.17±0.19	n.c.	n.c.	n.c.	n.c.
	19	-8.57±1.02	-0.32±0.19	n.c.	n.c.	n.c.	n.c.
Average		-6.00±1.02	-0.21±0.19	-2.77±1.05	-0.16±0.25	-7.42±0.97	-0.17±0.15
**Total**^f^		-5.26±0.99	-0.19±0.19	-2.48±1.01	-0.14±0.24	-6.86±0.78	-0.15±0.12

^a:^ MED—Mean elevation difference;

^b:^ SMB—Specific mass balance (m/a w.e.);

^c:^ n.a.—no data;

^d:^ n.c.—not calcualted as some portion of glacier was covered with cloud or more snow;

^e:^ Average values were calculated for all glaciers in each sub-region;

^f:^ Total values of all glaciers in the Muztag Ata-Kongur Tagh.

Glaciers in MAKT exhibited heterogeneous mass changes during 1970s–2014. Some glaciers showed stable or little change in their area, but down-wasting at the same time. For example, Koksay Glacier (No. 5), the largest glacier of Muztag Ata massif, thinned by 9.96 ± 0.65 m (–0.22 ± 0.10 m w.e. a^–1^) during 1976–2014. The mass changes varied from glacier to glacier in different periods. Glacier G075247E38305N (No. 10) showed a negative mass change (–0.47 ± 0.18 m w.e. a^–1^) before 1999 and a nearly balanced state (0.01 ± 0.23 m w.e. a^–1^) after 1999. Karayaylak Glacier (G075254E38623N), the largest glacier of Muztag Ata and Kongur Tagh, exhibited a mass loss (–0.32 ± 0.19 m w.e. a^−1^) during 1976–1999; however, the mass budget of Karayaylak Glacier was positive (0.37 ± 0.25 m w.e. a^−1^) after 1999.

**Fig 3:**
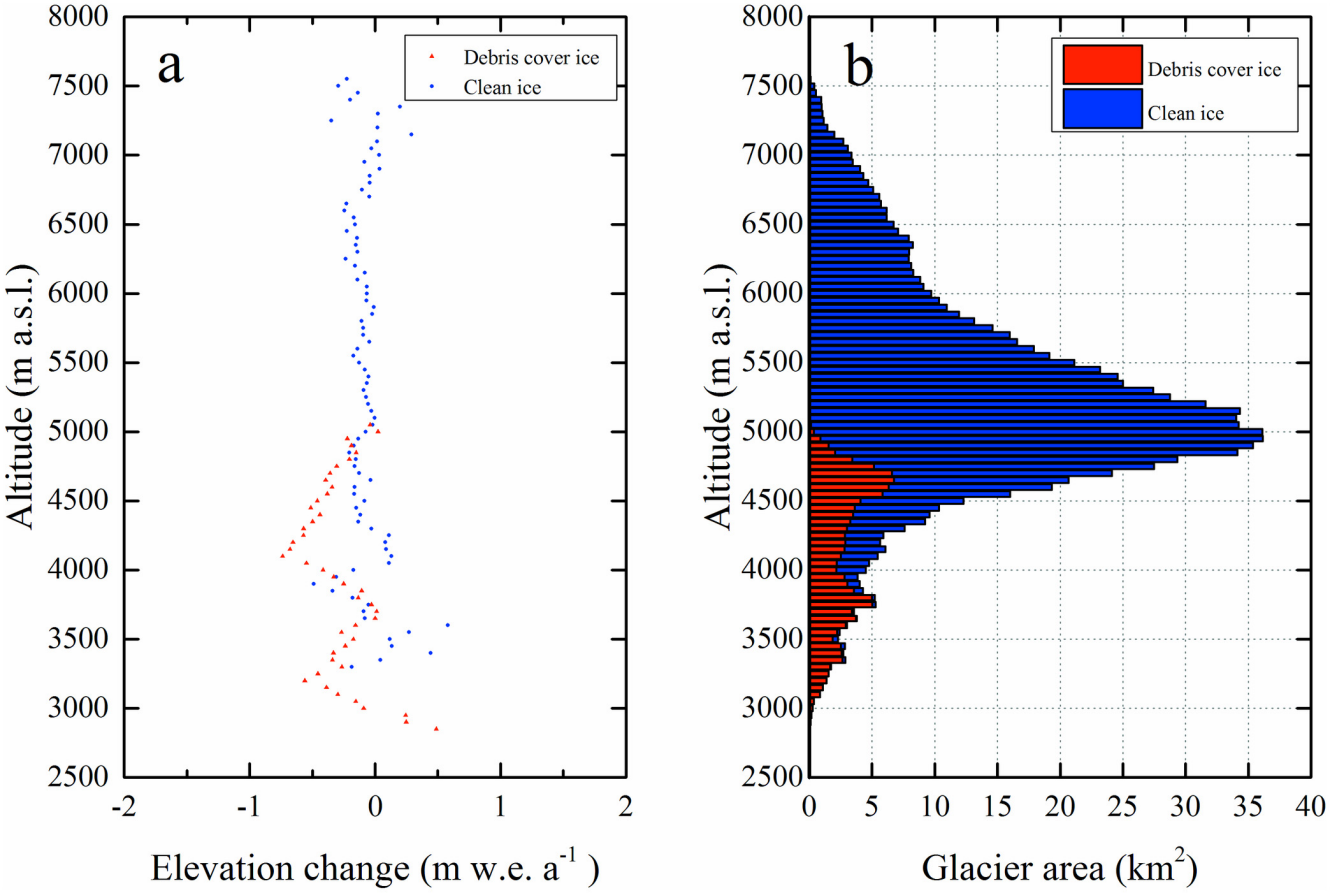
(a) Altitudinal distribution of the mean annual ice mass changes average at 50 m elevation bands for the clean-ice (blue) and the debris-covered (red) parts during 1971/76–2013/14; (b) Hypsometry of the glacier area in 2014 at MAKT.

The debris-covered glaciers showed stronger mass loss than the clean ice. Their mass budget was –0.32 ± 0.12 m w.e. a^−1^ for the period 1971/76–2013/14 (Fig 3) and thinning was especially obvious for the region from 4000 to 4500 m a.s.l. with a mass budget of –0.54 ± 0.12 m w.e. a^−1^ on average. Over the debris-covered glacier around 3500–4000 m a.s.l. there was a smaller mass change of –0.14 ± 0.12 m w.e. a^−1^. In the area on the glacier below 3500 m a.s.l., mass change was in the range –0.32 ± 0.12 m w.e. a^−1^ with spatially inhomogeneous surface ablation (Fig 3).

## Discussion

### Glacier area change

Muztag Ata and Kongur Tagh showed the least glacier shrinkage (~–1.9% during 1971/76–2014) compared with the other regions in the eastern Pamir [19], which may be attributed to the larger average glacier size, clustering, and relatively high elevation in this study region. The three largest glaciers (total area about 283.3 km^2^) exhibited a decrease of only ~0.1% (0.3 km^2^) from 1976 to 2014, and the total glacier area in Muztag Ata and Kongur Tagh increased by ~0.02% (0.18 km^2^) from 2009. Overall, large glaciers retreat slower than small ones [19]. Increased winter precipitation is probably the major reason for slight glacier expansion in recent years [13], which is consistent with the report of Yao, Thompson [10].

A consensus of glacier retreat measured in their area was commonly found in previous studies for glaciers in MAKT, but with various rates from –0.6% to –7.9% in different periods [11, 15, 16]. Our results lie within the range by Cai, Ma [15], Shangguan, Liu [16], and Holzer, Vijay [11]. We attributed the discrepancies of all the studies to their different study extent of glaciers and periods with available data. In addition, our result of glacier area change during the same time span in Muztag Ata (~–2.6 ± 0.4% during 1971/76–2014) approximates to that (–0.6 ± 3.9%) of Holzer, Vijay [11]. The difference of glacier changes from different authors might also relate to the inclusion/exclusion of steep headwalls as a part of the glacier. The resolution of Landsat imageries is lower than that of Pléiades, but the boundaries in our glacier mapping were verified through high-resolution images from Google Earth and available field GPS points (cf. [18, 59]). The steep headwalls in the accumulation area were not excluded in our glacier inventories assuming that they did not change during the considered period and they provide mass through ice/snow avalanches. This helps explain the difference between our study and that of Holzer et al. about glacier area for some glaciers. For example, the area of Koksay Glacier (No. 5), the largest glacier at Muztag Ata, was different by 81.2 ± 1.41 km^2^ in our study from 54.5 ± 0.11 km^2^ in Holzer, Vijay [11] (named as Kekesayi Glacier by Holzer, Vijay [11]).

Glaciers in the region might be weak retreating ones if compared with those in other mountain ranges in High Mountain Asia [10, 73]. The most comparable one may be found in West Kunlun Shan [6] where the glacier area decreased by 3.2 ± 3.1% (–0.16 ± 0.10% a^–1^) during the 1970s–1990 and 0.2 ± 2.5% (0.01 ± 0.32% a^–1^) during 1990–2010s. No significant area changes were reported for Karakoram [74–76]. The glacier area change value of –3.7 ± 4.8% (or –0.11 ± 0.14% a^–1^) (1975–2008) for central Tian Shan [77] is also similar to our result; however, a slightly higher shrinkage was recorded for the Sary-Djaz catchment [78] and western Chinese Tian Shan [79], and considerably greater shrinkage was observed for the other regions of Tian Shan, e.g., –0.6% a^–1^ in the Ili River basin [60].

### Glacier mass change

By integration of our result with that from Holzer, Vijay [11], we conclude that glaciers in the MAKT region have been in a weak mass loss trend. However, the mass loss values for glaciers in our study might be slightly higher than Holzer, Vijay [11] (–0.01 ± 0.30 m w.e. a^−1^ during 1973–2013 or –0.03 ± 0.33 m w.e. a^−1^ during 1973–2009) despite such uncertainties. Most results for glaciers in Muztag Ata might vary from Holzer and others. For example, the Koksay Glacier mass loss value (–0.22 ± 0.10 m w.e. a^−1^ during 1976–2014) might be higher than Holzer and others (–0.08 ± 0.30 m w.e. a^−1^ during 1973–2013 or –0.11 ± 0.33 m w.e. a^−1^ during 1973–2009). We consider that accuracies of different DEMs and GCPs may be the main reason for these deviations. The accumulation regions showed high noise from optical data with a high uncertainty for elevation, which was also subject to such eventual biases. However, the trends in glacier mass change in the past 40 years were consistent between us and Holzer and others. The trend biases after 1999 may contribute to uncertain penetration of SRTMs C-band radar into ice and snow, which also may explain why there are discrepancies with Gardner, Moholdt [8] (–0.13 ± 0.22 m w.e. a^−1^), Kääb, Treichler [9] (–0.48 ± 0.14 m w.e. a^−1^), and Gardelle, Berthier [4] (+0.14 ± 0.13mw.e. a^−1^) for glaciers’ mass change in Pamir after 1999 despite different study extents.

Early glacier surveys in the Muztag Ata verify that glacier ablation was intensified by atmospheric warming from 1960/87 to 2001 on Kematulejia Glacier about 4460–4600 m a.s.l. in the summer season [29]. Hydrological analysis based on the river runoff at Bulungkol (also called Kalakuli) hydrology station (74° 58′ W, 38° 40′ N, 3300 m a.s.l.) gives the result that glaciers in the Kangxiwa river basin in our study area during 1960–1990 lost –0.12 m w.e. a^−1^ [80], which is close to our result, and ice-core data from Muztag Ata Peak (75° 06′ E, 38° 17′ N, 7010 m a.s.l.) recorded a decrease in annual accumulation from 1958 to 2003 while a mass balance reconstruction showed a dramatic increase in wastage from 1990 to 2003 (–0.42 m w.e. a^−1^) [27]. Thus, under the condition of global warming, there is no doubt that glacier ablation has continually increased. At the same time, if the snow accumulation that develops glaciers had decreased, the mass balance would be strongly negative [81]. However, a positive balance was observed on the Muztag Ata glacier (No. 6) in recent years determined by higher snow accumulation with increasing precipitation from the strengthening westerlies [10].

One of the notable results is that the debris-covered regions obviously exhibit higher thinning rates with an average of –0.32 ± 0.12 m w.e. a^−1^ in our study area from the 1970s to 2013/14, although debris-covered area change was not significant. Early glacier surveys on Kematulejia Glacier about 4460–4600 m a.s.l. verify that glacier ablation on debris-covered ice was greater than on clean ice [29]. The mass-loss patterns for debris-covered ice are complicated by complex surface conditions with supraglacial ponds, ice cliffs, and heterogeneity of debris cover [82]. The higher value of mass loss for glaciers about 4000–4500 m a.s.l. may be attributed to thick debris or other surface conditions. The ice in the lower altitudes with higher temperature condition and weak insulation effect of thick debris are likely more sensitive to increasing summer temperatures [35].

## Conclusion

We used remote sensing datasets of ASTER images (2013/14), Cartosat-1 (2014), and Landsat in conjunction with SRTM data (2000), topographic maps (1971/76), and the first and second Chinese glacier inventories to investigate glacier area and mass changes of Muztag Ata and Kongur Tagh. Our results show that the overall area loss of Muztag Ata and Kongur Tagh was –39.4 ± 5.8 km^2^ from 1963 to 2014, which accounts for 3.8 ± 0.6% of its area in 1963. Muztag Ata and Kongur Tagh were reduced by an area of –30.0 ± 4.7 km^2^ (–2.7 ± 0.5%) between 1971/76 and 2014. The glacier shrank slightly after 2000 (–2.5 ± 0.5 km^2^, or –0.2 ± 0.1%) and increased during the period 2009–2014 (0.5 ± 0.1 km^2^, or 0.1 ± 0.0%). The mass budget of MAKT was –0.19 ± 0.19 m w.e. a^−1^ for the period ~1971/76–1999 and –0.14 ± 0.24 m w.e. a^−1^ during 1999–2013/2014. Overall, Muztag Ata and Kongur Tagh lost –7.07 ± 0.80 km^3^ in ice volume (equivalent to –0.15 ± 0.12 m w.e. a^–1^) from 1971/76 to 2013/14. These losses are heterogeneous and differ spatially as well as temporally. Although debris-covered area change was not significant, the debris-covered regions obviously exhibit higher thinning rates, with an average of –0.32 ± 0.12 w.e. a^−1^ from the 1970s to 2013/14.
